# A European journal serving the world electrophysiological community: 25 years of EP Europace journal

**DOI:** 10.1093/europace/euad207

**Published:** 2023-08-25

**Authors:** Angelo Auricchio, Gerhard Hindricks, John A Camm, Richard Sutton

**Affiliations:** Division of Cardiology, Istituto Cardiocentro Ticino, Ente Ospedaliero Cantonale, Via Tesserete 48, CH-6900 Lugano, Switzerland; Deutsches Herzzentrum der Charité (DHZC), Department of Cardiology, Angiology and Intensive Care Medicine, Charitéplatz 1, D-10117 Berlin, Germany; Department of Cardiology, Charité—Universitätsmedizin Berlin, Corporate Member of Freie Universität Berlin and Humboldt-Universität zu Berlin, Charitéplatz 1, D-10117 Berlin, Germany; Department of Cardiology, German Centre for Cardiovascular Research (DZHK), Partner Site, Berlin; Department of Cardiology, Cardiology Clinical Academic Group, Molecular & Clinical Sciences Institute, St. George’s University of London, Cranmer Terrace, London SW17 0RE, UK; Department of Cardiology, Hammersmith Hospital, National Heart & Lung Institute, Imperial College, Du Cane Road, London W12 0HS, UK

This year marks the 25 years of publication of EP Europace—European Heart Journal (EHJ) Arrhythmia and Electrophysiology journal. To celebrate this notable achievement with the deserved emphasis, the journal Editorial board commissioned a large group of Editors to contribute a series of state-of-the-art reviews, which presents the historical overview and key scientific contributions of the journal to the world community of cardiac electrophysiologists and to cardiologists and physicians interested in the diagnosis, management, and treatment of various arrhythmia disorders. The state-of-the-art reviews appearing in the journal issue of August and September represent the tribute to the different fields of cardiac pacing and electrophysiology to which Europace, now named EP Europace—EHJ Arrhythmia and Electrophysiology, have proudly contributed to shape over 2 decades.

Built on the legacy of the European Journal of Cardiac Pacing and Electrophysiology (1992–97), the Europace journal was founded in 1998 by Prof. Richard Sutton.^1^ It represented a major commitment by the European Society of Cardiology to the publication of excellent scientific work from Europe and also from the rest of the world. The journal was the first specialty journal of the Society. The journal mission was to provide the scientific community with high-quality original science, opinion, and education from leading European and international authors covering the growing cardiac pacing and clinical electrophysiology field. The Working Groups on Cardiac Arrhythmias, Pacing, and Cardiac Cellular Electrophysiology committed to supporting the journal. To this end, Editorial Board members were drawn from all three Working Groups, and a very selected number of international experts were requested to contribute.

The first issue of the journal appeared on January 1999. It included reports from a study group comprising experts in cardiac pacing and electrophysiology on the management of patients with atrial fibrillation, which, at that time, was limited to cardiac pacing and eventually to the ablation of the atrioventricular node.^2^ An additional contribution included the study design of a landmark randomized controlled trial—the SAFE PACE 2—investigating the role of cardiac pacing in older patients with falls and carotid sinus hypersensitivity.^3^

Starting in 2007, the journal editorial activities were managed by Prof. John A. Camm. Under his leadership (until 2018), then Prof. Gerhard Hindricks (from 2018 until 2022) and now Prof. Angelo Auricchio, the international journal readership has witnessed impressive growth in the contribution of the journal to various developments in the field of wearable and implantable cardiac rhythm monitoring devices, catheter ablation for atrial fibrillation, ventricular arrhythmias, and most recently of cardioneuroablation, in cardiac devices including cardiac resynchronization therapy and cardiac conduction system pacing, sudden cardiac death, cardiac imaging, and cardiac genetics, just to mention a few.

Being the official journal of the European Heart Rhythm Association (EHRA), EP Europace—EHJ Arrhythmias and Electrophysiology represents the main communication platform for our Association. This year, the journal has already published about eight EHRA scientific documents and EHRA-related scientific initiatives such as EHRA surveys. EHRA scientific documents developed by EHRA Scientific Document Committee alone or in collaboration with other internationally recognized scientific organizations are a very important source of current knowledge in the field; they provide state-of-the-art treatment standards and recommendations. Furthermore, they place upcoming technologies and novel research avenues in the proper scientific framework. An excellent and most recent example of clinical practice-oriented scientific document is represented by the EHRA clinical consensus statement on conduction system pacing implantation: endorsed by the Asia Pacific Heart Rhythm Society, Canadian Heart Rhythm Society, and Latin American Heart Rhythm Society with an Altmetric score of 216 and an impressive number of views (over 23 000) in <6 weeks since its publication.^4^ An important recent initiative is represented by patient surveys. The findings of a prospective, multi-centre, and multinational European Heart Rhythm Association patient Survey ‘Living with an ICD’ included patients already implanted with an implantable cardioverter-defibrillator (ICD) have been recently published.^5^ An online questionnaire was completed by patients invited from 10 European countries. This represents a novel approach to addressing regional differences in patients’ quality of life and the provision of information; it provides an opportunity to expand the journal readership to patient organizations and national regulatory bodies.

Over time, the journal has constantly adapted its publication model, becoming on 1 January 2023 a full Open Access journal. This significant development further expands the international visibility of each accepted article including developing countries or geographies with limited access to science.

Each of the four Editorial Boards has progressively re-designed the manuscript handling process, aiming to meaningfully reduce the turnaround time of each submitted manuscript while ensuring a fair and balanced peer-review process. Most recently, to ensure a nearly immediate sharing with the scientific community of the original content of an accepted manuscript, starting on 15 June 2023, the author’s accepted manuscripts have been published as online PDFs within a couple of days after the author signs the publishing licence. A digital object identifier has been given to each manuscript, which allows immediate access to the work, article citation, and eventually a significant chance and opportunity for the transmission of messages and dissemination of article-related data within connected communities. Also, the article categories and manuscripts requirements have been steadily adapted to the changing needs of the scientific community. Most recently, several additional manuscripts categories have been added, e.g. ‘Fast Track’, ‘Controversy’, ‘Research letter’, ‘Trial design’, and finally ‘Practical EP’ articles. Finally, a major novelty of this year has been the non-acceptance of case reports, favouring the submission of highly educational, valuable case reports, images, and quality improvement projects to the ‘European Heart Journal—Case Reports’, another title within the rich ESC family of journals.

**Figure 1:**
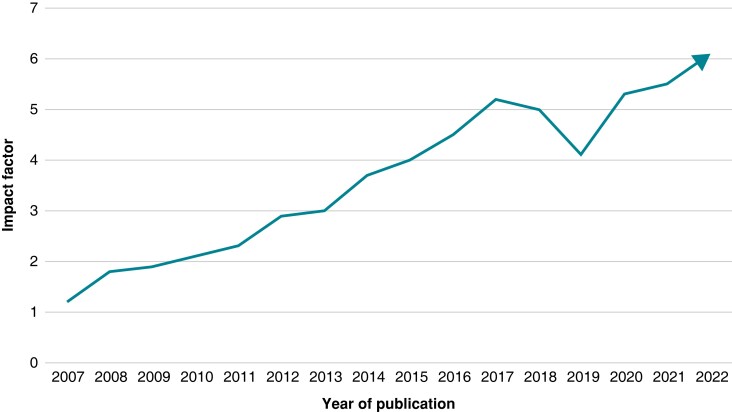
EP Europace—EHJ arrhythmias and electrophysiology journal impact factor growth over the past 2 decades.

Over 25 years, the journal enjoyed an impressive, steady increase in its impact factor which is now 6.1 thus, being the third in the specialty field of arrhythmias and electrophysiology (*Figure 1*), and the 29th out of 210 journals in the field of cardiac and cardiovascular systems.

This impressive development would not have been possible without the passion, dedication, and enthusiasm of the many who as authors, editors, reviewers, or readers have supported EP Europace—EHJ Arrhythmias and Electrophysiology, and we profoundly hope they will continue to do so in the future. This is also the right time to express our most heartfelt thanks to all members of the journal Editorial Board for accepting the invitation to devote their time to advising each of the past and present Editors-in-Chief and in performing invaluable editorial activities. The entire OUP editorial team must also be acknowledged for their unrestricted commitment to the progress of the journal.
